# Alternative Polyadenylation and Differential Regulation of *Ucp1*: Implications for Brown Adipose Tissue Thermogenesis Across Species

**DOI:** 10.3389/fped.2020.612279

**Published:** 2021-02-09

**Authors:** Wen-Hsin Lu, Yao-Ming Chang, Yi-Shuian Huang

**Affiliations:** Institute of Biomedical Sciences, Academia Sinica, Taipei, Taiwan

**Keywords:** alternative polyadenylation, brown adipose tissue, thermogenesis, transcriptional control, translational control, uncoupling protein 1

## Abstract

Brown adipose tissue (BAT) is a thermogenic organ owing to its unique expression of uncoupling protein 1 (UCP1), which is a proton channel in the inner mitochondrial membrane used to dissipate the proton gradient and uncouple the electron transport chain to generate heat instead of adenosine triphosphate. The discovery of metabolically active BAT in human adults, especially in lean people after cold exposure, has provoked the “thermogenic anti-obesity” idea to battle weight gain. Because BAT can expend energy through UCP1-mediated thermogenesis, the molecular mechanisms regulating UCP1 expression have been extensively investigated at both transcriptional and posttranscriptional levels. Of note, the 3′-untranslated region (3′-UTR) of *Ucp1* mRNA is differentially processed between mice and humans that quantitatively affects UCP1 synthesis and thermogenesis. Here, we summarize the regulatory mechanisms underlying UCP1 expression, report the number of poly(A) signals identified or predicted in *Ucp1* genes across species, and discuss the potential and caution in targeting UCP1 for enhancing thermogenesis and metabolic fitness.

## Introduction

Brown and white adipocytes possess unique functions in thermogenesis and energy storage, respectively; however, to some extent, one could cover the function of the other. A high fat diet (HFD) can induce brown-to-white adipocyte conversion when brown adipocytes store excess lipids to appear unilocular. Some white adipocytes are known as beige adipocytes, which can be browned in response to adrenergic signaling to become thermogenic (1). Cold-induced metabolic activity of brown adipose tissue (BAT) is positively correlated with resting metabolic rate and negatively with body mass index and body fat percentage in humans (2, 3). Therefore, a “thermogenic anti-obesity” approach has been proposed to combat obesity if the metabolic furnace, BAT, can be pharmacologically activated to burn excess fat (4). Because BAT defends against fat accumulation *via* uncoupling protein 1 (UCP1)-mediated thermogenesis, extensive efforts have uncovered how UCP1 activity is controlled by allosteric conformation changes and gene expression to affect thermogenesis. Several polymorphisms in the promoter, non-coding, and coding regions of *UCP1* gene were found associated with obesity and type 2 diabetes (5–8). Of note, genomic variations in *Ucp1* across species indicate that non-shivering thermogenic function and regulation are not necessarily conserved through evolution.

### Discovery of *Ucp1*, Whose 3′-UTR Is Processed Differently Among Species

**Figure 1:**
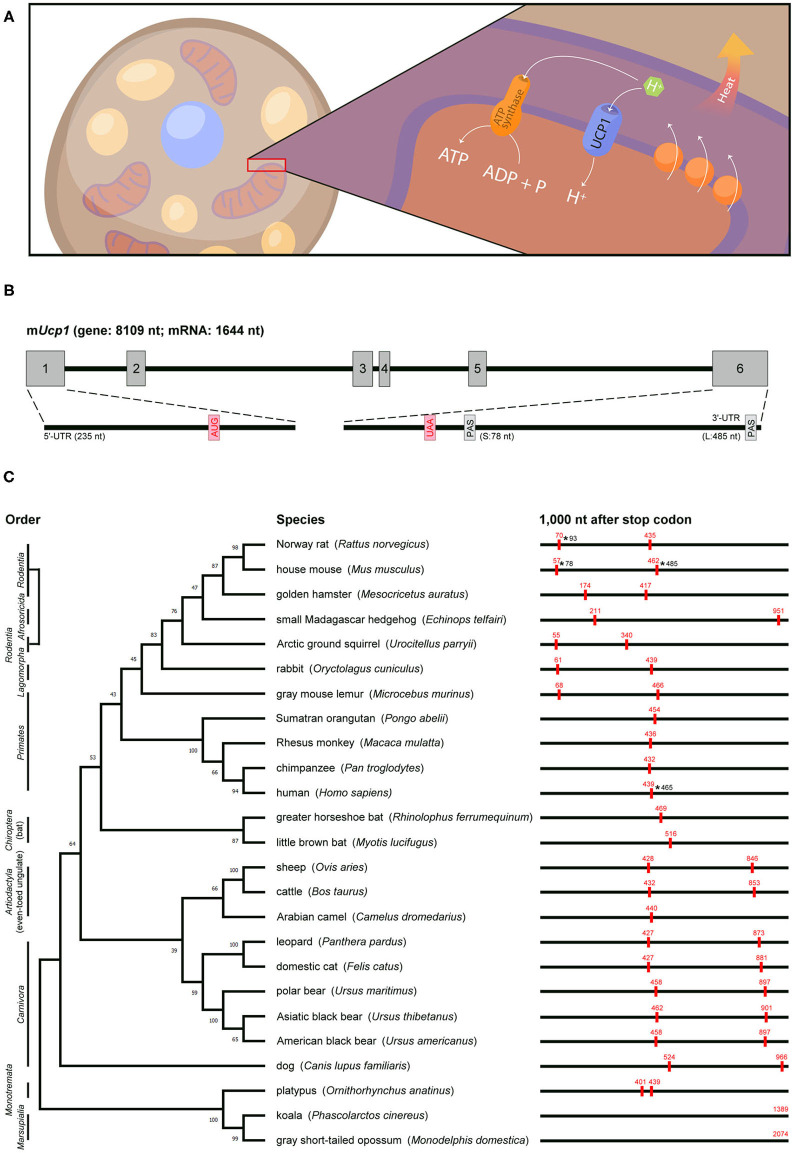
Genomic information, actual or predicted polyadenylation sites in *Ucp1* across species. **(A)** UCP1, a proton channel located in the inner mitochondrial membrane of brown adipocytes, transports protons to the matrix and then disrupts the proton gradient generated by the electron transport chain, thus uncoupling fuel oxidation from ATP synthesis and releasing energy as heat. **(B)** Mouse *Ucp1* spans ~8 kb and contains six exons (gray boxes). The start (AUG) and stop (UAA) codons are labeled in red, and the two polyadenylation sites (PAS) are denoted. The 5′-UTR and 3′-UTR of *Ucp1* are located on exons 1 and 6, respectively. **(C)** Phylogenetic relationship of mammalian *Ucp1* by alignment of the nucleotide sequences of coding regions. Animal orders are marked in the left and the percentage of bootstrap support of minimum evolution is shown on the corresponding branch. The BLAST search of 1,000 nt after the *Ucp1* stop codon across species involved the Ensembl or NCBI database. The poly(A) signals (AAUAAA) are marked as red boxes with their positions in red. The ends of 3′-UTRs confirmed by cDNA sequencing in rat, mouse, and human are labeled with asterisks and numbers.

UCP1 was first isolated from BAT of rats and hamsters in 1980 and named for its heat-producing function by uncoupling the oxidative phosphorylation process in mitochondria (Figure 1A) (9). At the basal status, the binding of purine nucleotide di- and tri-phosphates on the outer facing cavity of UCP1 blocks its proton-translocating activity (10, 11). In response to nutrients or cold environment, thermogenesis is activated *via* the release of norepinephrine from sympathetic circuits onto β3 adrenergic receptors (β3ARs) in brown adipocytes (4, 12, 13). β3AR signaling elevates cyclic adenosine monophosphate (cAMP) level to activate protein kinase A and lipolysis and acutely enhances UCP1 activity *via* free fatty acid-induced allosteric changes (14). Chronic activation of β3ARs promotes UCP1 synthesis by multiple transcriptional and posttranscriptional mechanisms to be discussed later.

Rat and mouse *Ucp1* cDNAs were cloned in 1985 (15, 16) and later sequenced to reveal their coding sequences and untranslated regions (UTRs) (17–19). Soon after, the rabbit *Ucp1* cDNA sequence was reported (20). The 3′-UTR of *Ucp1* in all three species contains two poly(A) signals (i.e., AAUAAA). Northern blot analysis revealed two *Ucp1* transcripts of ~1.5 and 1.9 kb (hereafter called *Ucp1S* and *Ucp1L*) in mouse and rat BAT (15, 16). The transcripts result from an alternative use of the poly(A) signal to yield 3′-UTRs of 78 and 485 bp in mice, respectively (21) (Figure 1B). By contrast, only the distal poly(A) signal is used to generate *Ucp1L* in rabbit BAT (20). The human *UCP1* coding sequence was identified by screening a genomic library with a rat *Ucp1* cDNA probe, and a 1.9-kb *UCP1* mRNA was detected in human perirenal BAT (22). More recently, the sequence of human *UCP1* cDNA (ENST00000262999) revealed only one poly(A) site in the 3′-UTR, and RT-PCR analysis confirmed that human *UCP1* encodes only the long form carrying a 465-bp 3′-UTR (21).

### Thermogenic UCP1 Is Not Preserved in All Endothermic Placental Mammals

The functional *Ucp1* gene consists of six exons and spans a region of ~8–10 kb among species (an example of mouse *Ucp1* is in Figure 1B). The phylogenetic relationship of *Ucp1* across species has revealed that the non-thermogenic UCP1 in fish and frogs (23) was derived from a hypothetical proto-UCP hundreds of millions of years ago. Non-thermogenic UCP1 was first lost in Sauropsida (reptiles and birds) around 300 million years ago, then thermogenic UCP1 emerged in endothermic placental mammals about 100 million years ago (24, 25). Although UCP1-mediated non-shivering thermogenesis is believed to provide a survival advantage in the cold and retain the body temperature of eutherian neonates after birth, eight mammalian clades, namely Xenarthra, Pholidota, Cetacea, Sirenia, Proboscidea, Hyracoidea, Equidae, and the most well-known Suidae (pigs), showed inactivation of *Ucp1* (26). The loss of exons 3 to 5 in pig *Ucp1* occurred ~20 million years ago, so pigs produce no functional UCP1 and depend on shivering for thermoregulation (27).

The previous studies aligned the genomic or amino acid sequences of *Ucp1* across species to build up the phylogenetic tree and derive an evolutionary timeline for *Ucp1* (24–26). We followed these evolutionary patterns by aligning the nucleotide sequences of the *Ucp1* coding regions in selected mammals and analyzed whether alternative polyadenylation could follow any evolutionary path (Figure 1C). The phylogenetic tree of *Ucp1* grouped most species following the canonical taxonomy, classifying animals of the same order in the same branch. The three exceptions are *Ucp1* in the small Madagascar hedgehog in *Afrosoricida* more related to rodents, in the gray mouse lemur in *Primates* more related to rodents and rabbits, and in the dog quite different from other animals in *Carnivora* (24).

The 3′-UTR information for *Ucp1* cDNAs in various species is often lacking or incomplete. We predicted “alternative polyadenylation” by searching the poly(A) signal (i.e., AATAAA) within 1 kb downstream of the stop codon from *Ucp1* genomic sequences because the 3′-UTR, including in mouse and human *Ucp1* mRNAs, is limited in the last coding exon of most transcripts. Our analyses revealed many representative animals carrying two poly(A) sites, such as mice, rats, and squirrels in *Rodentia*; rabbits in *Lagomorpha*; and cats, dogs, cows, leopards, and bears in *Laurasiatheria*. However, microbats and greater horseshoe bats in *Chiroptera* are the exceptions, with only one poly(A) motif in the 3′-flanking region. Notably, almost all primates, such as humans, chimpanzees, orangutans, and macaques, have only one poly(A) site in *Ucp1*, with the exception of mouse lemurs, which carry two poly(A) signals at the position similar to those of rodent *Ucp1*. The nucleotide sequence of the *Ucp1* coding region in mouse lemurs was unexpectedly more similar to those in rodents than primates (Figure 1C). Although the platypus, a unique placental mammal, has two adjacent poly(A) signals in *Ucp1*, alternative polyadenylation, even if it occurs, would generate two 3′-UTRs of only 32 bp difference. The poly(A) site of *Ucp1* in koala and gray short-tailed opossum in *Marsupialia* is located at 1,389 and 2,074 bp downstream of the stop codon, respectively, implying *Ucp1* in both species may be subjected to more complex posttranscriptional regulation. Other than the 3′-UTRs of rat *Ucp1S*, mouse *Ucp1S* and *Ucp1L*, and human *UCP1* having been confirmed by cDNA sequencing (17, 18, 21), the actual usage and preference of the two putative polyadenylation sites in other species require further investigation.

### Transcriptional Regulation of *Ucp1* Gene

**Figure 2:**
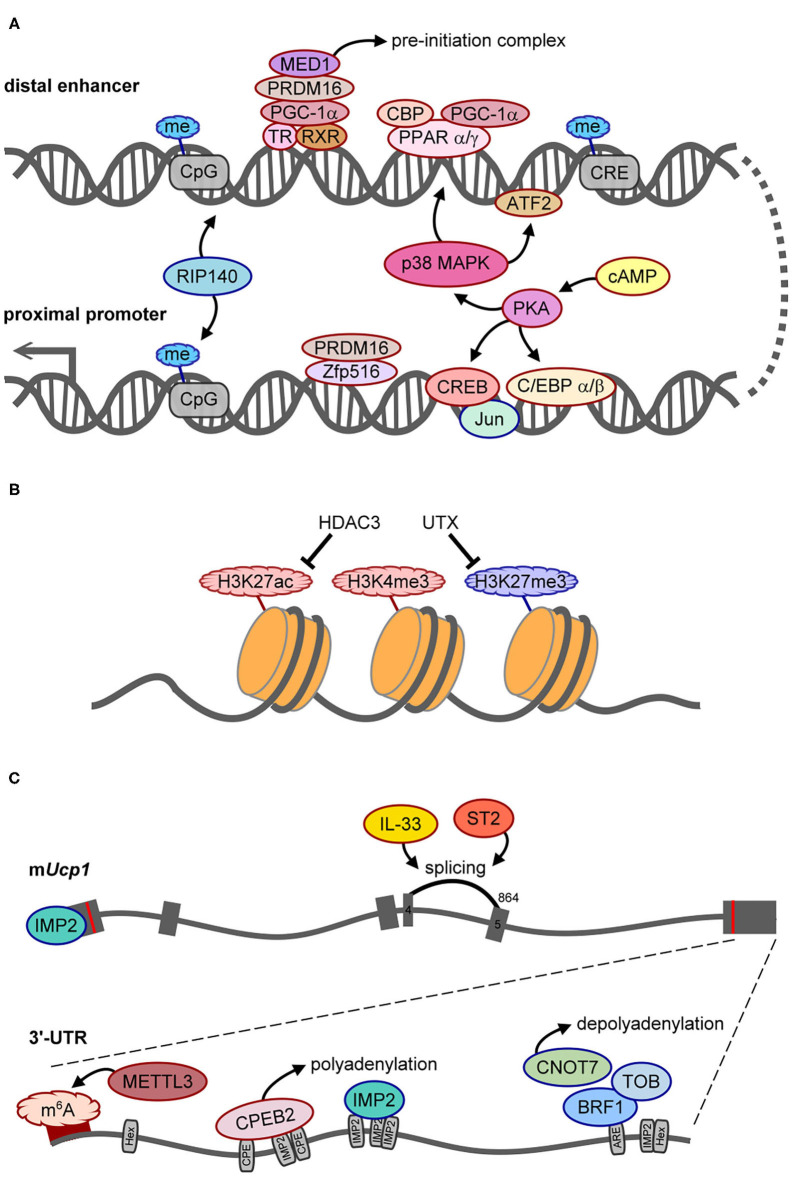
The molecular mechanisms control UCP1 synthesis. Positive and negative regulators of UCP1 expression are outlined in red or blue, respectively. **(A)** Scheme of regulatory factors acting on the distal enhancer and proximal promoter of *Ucp1*. **(B)** The histone modifications, HDAC3 and UTX demethylase, involved in epigenetic regulation of *Ucp1* transcription. **(C)** Illustration of splicing and posttranscriptional regulation of *Ucp1* RNA. The gray boxes are six exons of *Ucp1* with the start (AUG) and stop (UAA) codons labeled as red lines. The number 864 above exon 5 is the splice acceptor site for generating functional UCP1. The bottom gray line represents the 3′-UTR of mouse *Ucp1* with 2 hexanucleotides (Hex, AAUAAA polyadenylation site), denoted regulatory sequences, including CPE (CPEB2-binding site), ARE (AU-rich element for BRF1 binding), and predicted IMP2-binding sites. The dark red band indicates the hot spot area for m^6^A modification.

Cold exposure markedly increases both *Ucp1L* and *Ucp1S* mRNA levels in mouse and rat BAT (15, 16), so elaborative efforts have identified the molecular mechanisms controlling *Ucp1* transcription (28–30). DNA footprinting and promoter assays were performed to identify a 212-bp distal enhancer (−2,494 to −2,283 bp from the transcription start site) and a 555-bp proximal promoter (−611 to −57 bp) in the 5′ non-coding region of rat *Ucp1* gene (28, 31). Many studies used *in vitro* assays to identify the *trans*-acting factors for *Ucp1* transcription, including protein kinase A-activated cAMP-response element binding protein (CREB) to compete with the negative regulator, Jun, for binding to the −139- to −122-bp region, and CCAAT/enhancer-binding protein α and β (C/EBPα and β) to activate through regions −457 to −440 and −335 to −318 bp [for details, see the review (32)]. Zfp516 binds to the promoter region (−70 to −45) with PR domain-containing 16 (PRDM16), which is essential for BAT differentiation and promotes browning of beige adipocytes to increase body temperature and energy expenditure (33) (Figure 2A). Notably, the absence of the CCAAT sequence in the human *UCP1* promoter suggests evolutionary variations in regulating *Ucp1* transcription (22).

When fused to a thymidine kinase promoter-driven reporter in a transgenic mouse line, the 212-bp enhancer sufficiently conferred BAT-specific expression and cold- and β3AR agonist-induced expression (34). Peroxisome proliferator-activated receptor α and γ (PPARα and PPARγ), PPARγ-coactivator 1α (PGC-1α), and CREB-binding protein (CBP) bind to the enhancer (−2,485 to −2,458) together to upregulate *Ucp1* transcription (35) (Figure 2A). Phosphorylation of PGC-1α and activating transcription factor 2 (ATF2) by cold exposure-activated p38 mitogen-activated protein kinase can directly or indirectly potentiate *Ucp1* transcription because *Ppargc1a* (i.e., the gene name of PGC-1α) transcription is also enhanced by phosphorylated ATF2 (35, 36). Moreover, PRDM16 recruited by PGC-1α interacts with thyroid receptor/retinoid X receptor heterodimers, and MED1, a component of the Mediator complex, is then recruited by PRDM16 to form a pre-initiation complex on the enhancer to facilitate *Ucp1* transcription (37, 38) (Figure 2A). Therefore, the enhancer region of *Ucp1* is regulated by signaling of multiple *trans*-acting factors through β3ARs.

In addition to positive regulators, receptor-interacting protein 140 (RIP140) mediates methylation at CpG sites in both the promoter and enhancer to silence *Ucp1* transcription (39) (Figure 2A). In pregnant mice with streptozotocin-induced diabetes, intrauterine exposure to hyperglycemia impaired fetal BAT development by altering methylation in several metabolic genes including *Ucp1*. Such epigenetic changes can last and affect BAT function and glucose homeostasis later in life (40). Besides CpG methylation, epigenetic modification of histone 3 (H3) affects chromatin structure to regulate transcription. Histone deacetylase 3 (HDAC3) controls the acetylation of H3 on lysine 27 (H3K27ac) in the enhancers of *Ucp1* and *Ppar*α (Figure 2B), so its deficiency facilitates *Ucp1* and *Ppar*α transcription and browning in beige adipocytes (41). Of note, pregnant mice fed omega-3 polyunsaturated fatty acids resulted in epigenetic changes, including decreased tri-methylation of H3 on lysine 27 (H3K27me3), increased H3K27ac and di-methylation of H3 on lysine 9 (H3K9me2) to affect BAT development, and increased *Ucp1* and *Ppargc1a* transcription in mouse offspring even at 8 weeks after post-weaning (42). H3K9me2 is usually defined as a repressed histone mark (43), so increased H3K9me2 may not directly associate with the *Ucp1* promoter. Nevertheless, this study reported that a maternal dietary effect could have long-lasting benefits on the development and function of the offspring's BAT. Moreover, the silencing mark, H3K9me2, was identified at the *Ucp1* enhancer in white adipose tissue (WAT), whereas the active mark, H3K4me3, at the *Ucp1* promoter was found in BAT in response to cold stimulation (43). The ubiquitously transcribed tetratricopeptide repeat on chromosome X (UTX) is a histone demethylase recruited to *Ucp1* upon the activation of β3ARs to reduce H3K27me3 level (Figure 2B), which consequently leads to increased H3K27ac level *via* unidentified histone acetyltransferases (44). Therefore, epigenetic changes on *Ucp1* are dynamically regulated for temporal and spatial control of UCP1 synthesis, and intrauterine nutrient exposure has a long-term effect on metabolic gene expression *via* epigenetic regulation.

### Posttranscriptional Regulation of *Ucp1* mRNA

A pre-mRNA undergoes several processing steps, including splicing, 3′-end cleavage, and polyadenylation, to become a mature mRNA. Alternative polyadenylation generates *Ucp1* mRNA carrying long (*Ucp1L*, ~10%) or short (*Ucp1S*, ~90%) 3′-UTR in mice and rats, but rabbits and humans express only the long form. The regulatory sequences in the 3′-UTR dictate posttranscriptional efficiency for protein production (45), but the effect of different *Ucp1* 3′-UTRs on UCP1 synthesis and thermogenesis has not been addressed until recently. The study of *Ucp1*Δ*L* mice, with 10% *Ucp1L* converted to *Ucp1S*, reported that the protein but not the mRNA level of UCP1 was reduced ~50–60% in *Ucp1*Δ*L* BAT, so *Ucp1L* is translated ~15 times more efficiently than *Ucp1S* in BAT (21). Moreover, the same study also identified that cytoplasmic polyadenylation element binding protein 2 (CPEB2) signaling through β3ARs binds to and activates polyadenylation-induced translation of only *Ucp1L* but not *Ucp1S* (Figure 2C), so both CPEB2-knockout (KO) and *Ucp1*Δ*L* mice show reduced UCP1 level and impaired BAT thermogenesis (21).

The other posttranscriptional mechanisms identified to date inhibit UCP1 synthesis. Reporter assay revealed that insulin-like growth factor 2 mRNA-binding protein 2 (IGF2BP2/IMP2) is a type 2 diabetes- and obesity-associated gene that suppresses *Ucp1* mRNA translation likely *via* both 5′- and 3′-UTRs. Thus, IMP2-null mice show increased energy expenditure, insulin sensitivity, and glucose tolerance and defend against cold temperature better than wild-type mice (46). HFD reduces the amount of UCP1 in obese adipose tissues by diminishing the stability of mouse *Ucp1* mRNA *via* the CCR4–NOT deadenylase complex. Moreover, HFD induces the expression of several subunits of the CCR4–NOT complex, including Cnot1–3, Cnot6 and Cnot7, and Cnot7-interacting Tob in inguinal WAT (iWAT) of obese mice. The binding of butyrate response factor 1 (BRF1) to the 3′-UTR of *Ucp1L* mRNA recruits Tob and Cnot7 to cause deadenylation-induced *Ucp1* decay (Figure 2C). Thus, Cnot7- or Tob-KO mice are resistant to HFD-induced obesity with increased *Ucp1* mRNA level in iWAT and BAT (47). Although IMP2 and BRF1 suppressed the translation and stability, respectively, of a reporter appended to the *Ucp1L* 3′-UTR in HEK293 cells, both studies did not examine *Ucp1L* and *Ucp1S* separately when comparing polysomal distribution (46) or mRNA stability of *Ucp1* (47) between wild-type and KO adipose tissues.

MicroRNAs (miRNAs) are a conserved class of small RNAs of about 20–22 nt that regulate the expression of their target mRNAs *via* the miRNA-induced silencing complex (48, 49). Despite the relatively short 3′-UTR (485 bp) of mouse *Ucp1L* mRNA, a previous study used miRanda to identify miR-9 and miR-338-3p as putative miRNAs targeting the 3′-UTR of *Ucp1L* mRNA (50). Activation of β3AR signaling decreased both miRNA levels and increased *Ucp1* mRNA level in epididymal WAT (eWAT) but not iWAT and BAT. Despite a negative correlation between miR-9 and miR-338-3p and *Ucp1* expression in eWAT, no further experiments were performed to determine whether both miRNAs affect *Ucp1* mRNA stability by binding to the predicted target sites in the 3′-UTR (50). Although cold exposure did not change the proportion of *Ucp1L* and *Ucp1S* in BAT (21), no studies have measured the proportion of *Ucp1L* and *Ucp1S* in WAT and whether it can be changed under HFD or cold temperature. The alternative use of proximal and distal poly(A) signals in mouse *Ucp1* mRNA may vary in different adipose tissues.

A recent paper demonstrated that methyltransferase like 3 (METTL3)-catalyzed m^6^A modifications in *Ucp1* mRNA increased its mRNA and protein levels during postnatal BAT development (Figure 2C). In mice with BAT-specific ablation of *Mettl3*, the loss of m^6^A-enhanced UCP1 expression reduced energy expenditure and cold tolerance (51). Moreover, these mice showed accelerated HFD-induced obesity, impaired glucose intolerance, and insulin resistance (51). Depending on the reader, m^6^A modification on mRNA can affect translation efficiency or mRNA stability (52), but how this epitranscriptomic modification mechanistically affects UCP1 expression has not yet been answered.

Besides these posttranscriptional regulations, interleukin 33 (IL-33) and ST2 (also known as IL-1 receptor 4, a receptor of IL-33) are required for accurate splicing of *Ucp1* mRNA between exons 4 and 5 by using the splice site at nucleotide 864 on exon 5 (Figure 2C). Without IL-33 or ST2, the splicing machinery prefers the cryptic nucleotide 972 site on exon 5 to produce a non-functional transcript (53). Depletion of either protein results in the loss of UCP1 in brown and beige adipocytes, so IL-33/ST2 signaling is important to register brown and beige adipocytes for uncoupled respiration and thermogenesis during the perinatal period (53).

## Discussion

Treating lean healthy men with a specific β3AR agonist, CL316243, increased insulin sensitivity and fat oxidation without affecting body weight (54), but 4-week administration of another β3AR agonist, L796568, in obese men did not confer evident metabolic benefits (55). The lack of chronic effect on shifting energy balance for weight loss may be due to few β3AR-responsive adipose tissues in humans. Indeed, two recent studies found a very low level of β3AR mRNA in human BAT (56, 57). Using cultured human brown adipocytes, one study reported that β2AR activation increased UCP1 level and lipolysis (56), but the other study identified β1AR signaling as responsible for upregulating thermogenic gene expression (57). Because the activation of β1AR and β2AR has a profound impact on cardiopulmonary function, the positive regulatory mechanisms downstream of β-adrenergic signaling must be targeted to promote UCP1 synthesis, energy expenditure, and metabolic fitness in humans. Notably, the mouse model has been used to reveal mechanisms controlling UCP1 synthesis, thermogenesis, and possible implications for human obesity. However, the loss of the CCAAT sequence and Sp1-binding motif in the proximal promoter (22) and alternative polyadenylation in human *UCP1* (21) supports that UCP1 expression is regulated by species-dependent variations. For example, despite comparable UCP1 activity in mouse and human BAT (58), we reported that the expression of human *Ucp1* mRNA in five different BAT samples is only 1/30–1/300 the level in mice (21). Our result agrees with a Northern blot study, which detected 1.9-kb *UCP1* mRNA by using 5 μg poly(A) RNA from human perirenal BAT. Thus, absolute RT-qPCR was used to compare the amount of *Ucp1* mRNA between human and mouse BAT (21). Because *Ucp1L* is translated 15 times more efficiently than *Ucp1S* in mouse BAT, we predicted that CPEB2-mediated translational activation, if fully functioning in human BAT, can make up the protein level to ~1/2–1/20 of that in mouse BAT (21). Similarly, if the mechanism of BRF1-mediated deadenylation-induced *Ucp1L* decay (47) is conserved in humans, it is expected that HFD-induced *UCP1* mRNA degradation in human beige adipocytes should become more prominent. Thus, humans, and perhaps also other primates, may rely more on posttranscriptional regulation to control UCP1 synthesis because they express a low level of only long 3′-UTR *UCP1* mRNA. Moreover, although ectopic expression of mouse *Ucp1* in white adipocytes of pigs also enhances thermogenic capacity and lipolysis to decrease fat deposition (59), why UCP1-mediated metabolic plasticity is lost in some mammals through evolution remains unclear. Therefore, the results from studying gene-modified mice in BAT thermogenesis need to be translated with caution into humans and other species.

## Author Contributions

W-HL wrote the manuscript and illustrated the figures. Y-MC analyzed the poly(A) sites in *Ucp1* across species. Y-SH co-wrote the manuscript and is responsible for its content. All authors contributed to the article and approved the submitted version.

## Conflict of Interest

The authors declare that the research was conducted in the absence of any commercial or financial relationships that could be construed as a potential conflict of interest.
